# Import Risk Analysis of the Decapod Iridescent Virus 1 (DIV1) Applied in South Korea: Qualitative Risk Review and Institutional Improvement Plans

**DOI:** 10.1155/2023/9504014

**Published:** 2023-08-12

**Authors:** Gyoungsik Kang, Won-Sik Woo, Kyung-Ho Kim, Ha-Jeong Son, Min-Young Sohn, Ju-Won Kim, Chan-Il Park

**Affiliations:** ^1^Department of Marine Biology and Aquaculture, College of Marine Science, Gyeongsang National University, Tongyeong 53064, Gyeongsangnam-do, Republic of Korea; ^2^Biotechnology Research Division, National Institute of Fisheries Science, Busan 46083, Republic of Korea

## Abstract

Decapod iridescent virus 1 (DIV1) is a dangerous new virus that causes mortality to shrimp and crayfish. This virus has been reported in China, which is a major exporter of decapods to South Korea and its neighboring countries, Taiwan and Thailand. This virus can be regarded as a pathogen with a high risk of entry into Korea. Therefore, South Korea has been conducting emergency quarantine for DIV1 since 2020, and in this study, an import risk analysis was performed following the World Organization of Animal Health and domestic laws and regulations to present scientific grounds and validity for South Korea's DIV1 emergency quarantine. Import risk analysis was performed by hazard identification, entry assessment, exposure assessment, and consequence assessment. As a result of the import risk analysis, it was confirmed that the current DIV1 quarantine can significantly reduce the import risk. However, it was also established that the management of prawns and polychaetes intended for animal feed or bait and currently not subjected to quarantine is also required. Additionally, two species of crabs that would meet the criteria for a completely susceptible species and two species of crayfish that required surveillance of the import amount or route of exposure were also identified. In conclusion, these uses and susceptible species were designated as quarantine targets for nonhuman consumption requiring risk management and import sanitary conditions to be performed before export and after import. Finally, alternatives to reduce the cost burden of stakeholders following the introduction of the system were also presented.

## 1. Introduction

Decapod iridescent virus 1 (DIV1) belongs to the family Iridoviridae and mainly affects juvenile shrimp and crayfish. It was first discovered in whiteleg shrimp (*Penaeus vannamei*) located in Zhejiang, China, in 2014 and named shrimp hemocyte iridescent virus in 2017 [1]. However, by 2018, the name was changed to DIV1 as the same virus was identified in shrimp farms in 11 provinces in China. In addition to DIV1, it is also called *Cherax quadricarinatus* iridovirus infection (CQIV), white head disease, and white spot disease [2–4]. The danger of this virus began to be known to ordinary fishermen through media reports in 2020 [5]. At the time of the media report in 2020, the infection rate and mortality rate were frighteningly high, and once the infection was confirmed, it was reported that it took only 2–3 days for all shrimp in the pond to die, and it has been reported as one of the dangerous diseases such as avian influenza and African swine fever [5].

DIV1 is a disease that can cause mortality to whiteleg shrimp (*P. vannamei*), one of the main cultured species in the nation, and its outbreaks have been reported in some areas of China and Taiwan so far [1, 2, 6–8]. South Korea currently imports many aquatic products (decapods) from China, the country where DIV1 is present, and its neighboring countries (Table 1). DIV1 is a disease listed on the “List of diseases in the Asia-Pacific” designated by the Network of Aquaculture Centers in Asia-Pacific (NACA), an Asia-Pacific regional consultative body [9], and in Australia, it is a “National list of reportable diseases of aquatic animals” as a disease subject to management in Australia, registered and managed [10].

South Korea designated DIV1 as an aquatic animal disease based on the risk information of DIV1, a new disease that occurred overseas in 2019 [8, 9, 11], conducted emergency quarantine, and notified trading countries of the measures through WTO/SPS (G/SPS/N/KOR/692, July 17, 2020). The quarantine measures implemented for DIV1, a novel disease that was not previously recognized by international standards, can be interpreted as a precautionary approach based on the sanitary and phytosanitary measures applied by other member nations. Therefore, the sanitary and phytosanitary measures need to be reviewed within a reasonable period (as soon as possible before an entry of a new disease) in accordance with Paragraph 5 of Article 5 of the WTO/SPS Agreement (*Risk Assessment and Determination of the Appropriate Level of Sanitary and Phytosanitary Protection*) by collecting additional necessary information for an objective risk assessment. In this respect, we conducted an import risk analysis to verify the scientific validity of the DIV1 quarantine measures through this study by collecting data such as scientific literature on DIV1.

DIV1 was listed as a World Organization of Animal Health (WOAH) designated disease in May 2021 (88th WOAH general session, 2021), but WOAH's Aquatic Code, Aquatic Manual, etc. do not contain detailed information about the disease. Therefore, through the DIV1 import risk analysis, it is necessary to analyze the risk assessment for each species subject to quarantine (including susceptible species and vectors), related imported product types (live, frozen, and refrigerated), information on available diagnostic methods, and the current status of domestic and foreign disease outbreaks. Through this analysis, it is necessary to review the DIV1 quarantine measures, which have not yet been reported in South Korea, and establish an effective management system.

## 2. Methodology

### 2.1. Background

When deemed necessary for an effective implementation of quarantine services in accordance with Article 37 (import risk analysis) of the Aquatic Organism Disease Control Act, the Minister of Oceans and Fisheries may assess the impact of the pathogens of aquatic organism diseases on the aquatic organisms and the aquatic environment when such pathogens are introduced to the nation, and conduct an analysis and evaluation (import risk analysis) on the import of aquatic organisms aimed to reduce the level of risk (Table 2). An import risk analysis should be conducted in the following order of the stages in accordance with the National Fishery Products Quality Management Service's Notification on the Methods and Procedures for the Implementation of Aquatic Organism Import Risk Analysis (Table 2).

### 2.2. Identification of Hazards (Aquatic Diseases)

The process of identifying and listing the types of pathogens that can potentially be introduced into South Korea due to the import of the specific aquatic organisms designated for quarantine purposes, etc. Regarding potential hazards (DIV1 in this study), (1) scientific literature and statistical data, (2) cases of other countries experiencing risks due to import routes and aquatic organisms (or their products) designated for quarantine purposes and pathogens, (3) experts in fisheries-related and import risk analysis opinion, and (4) aquatic organism disease pathogens designated by the WOAH.

The criteria for determining the risk factors are (1) relevance to aquatic organisms (or their products) designated for quarantine purposes, (2) distribution of susceptible species in South Korea and whether the species can cause serious diseases, and (3) whether they exist in exporting countries, and if present, risk factors are considered by the competent authorities of the exporting country manage the disease (reporting of disease outbreaks, operation of control and eradication systems). In addition, if the pathogen exists in South Korea, it was referred to in the selection of hazard factors by considering (1) if the pathogen in the exporting country is a highly pathogenic strain and (2) whether it exists in South Korea, an area where the pathogens do not occur or have a low level of occurrence. Finally, hazard identification was performed to determine whether DIV1 could potentially be introduced as a pathogen that negatively affects domestic aquatic life or aquatic ecosystems and the environment due to the import of species designated for quarantine purposes.

### 2.3. Risk Assessment of Aquatic Organism Diseases (Risk Assessment)

Qualitative assessment of the possibility of entry, settlement, and spread into the country of the hazard through the import of the aquatic organisms designated for quarantine purposes and the scale and influence of such effects on biological, environmental, economic, and other areas.

#### 2.3.1. Entry Assessment

**Figure 1:**
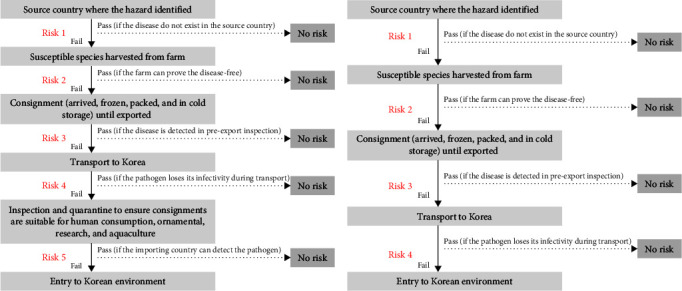
Entry pathway of designated quarantine products (a) and nonquarantine products (b).

The entry assessment is based on biological factors (breeds, strains, genotypes, age of aquatic organisms, strains of causative agents, infected or contaminated tissues, vaccination status, quarantine, etc.), national factors (incidence and prevalence in the exporting country, and capacity, surveillance and sanitation management program, evaluation of the localized system, etc.), and factors of aquatic organisms (or their products) designated for quarantine purposes (expected amount of import, ease of contamination, the impact of processing method on pathogens, impact of storage and transportation methods on pathogens, etc.). The entry assessment is performed according to the entry scenario, and the comprehensive entry risk is calculated based on the evaluation criteria for each scenario. The entry pathways and entry scenarios used in this study are illustrated and described in Figure 1 and Table 3.

#### 2.3.2. Exposure Assessment

**Figure 2:**
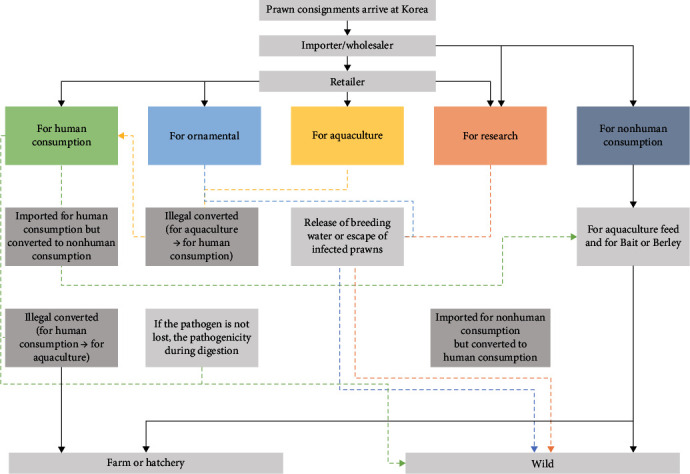
Exposure pathway and scenarios of DIV1.

Exposure assessment is also performed considering biological factors, national factors, and factors of aquatic organisms (or their products) designated for quarantine purposes. Biological factors include the genotype of the host, characteristics of the causative agent (toxicity, pathogenicity, survival variables, etc.), and the presence of potential vectors or intermediate hosts. For national factors, statistical data of aquatic organisms (the existence and distribution of susceptibility or vector species, etc.), geographical and environmental characteristics (aquatic geographic statistics, range of water temperature, etc.), and customs (cultural and consumptive) are considered. Finally, for the factors of aquatic organisms (or their products) designated for quarantine purposes, the expected amount of import, and the use of imported aquatic organisms or their products (distribution route, stockpiling method, aquaculture feed, use as bait, waste disposal method, etc.) are considered. Exposure assessment is also conducted according to exposure pathways and scenarios, and the literature and field investigations are conducted to confirm the number of all possible cases (Figure 2).

#### 2.3.3. Consequence Assessment

The consequence assessment is conducted considering direct and indirect results. As for the direct results, infection susceptibility and mortality of aquatic organisms, reduction in production, impact on the aquatic ecosystem, closure of related businesses, negative and irreversible impact on the environment, and impact on public health are considered. Indirect results are performed by considering surveillance and control costs, compensation costs, potential trade losses, negative consumer reactions, job loss, and the decrease in tourists.

#### 2.3.4. Risk Estimation

**Figure 3:**
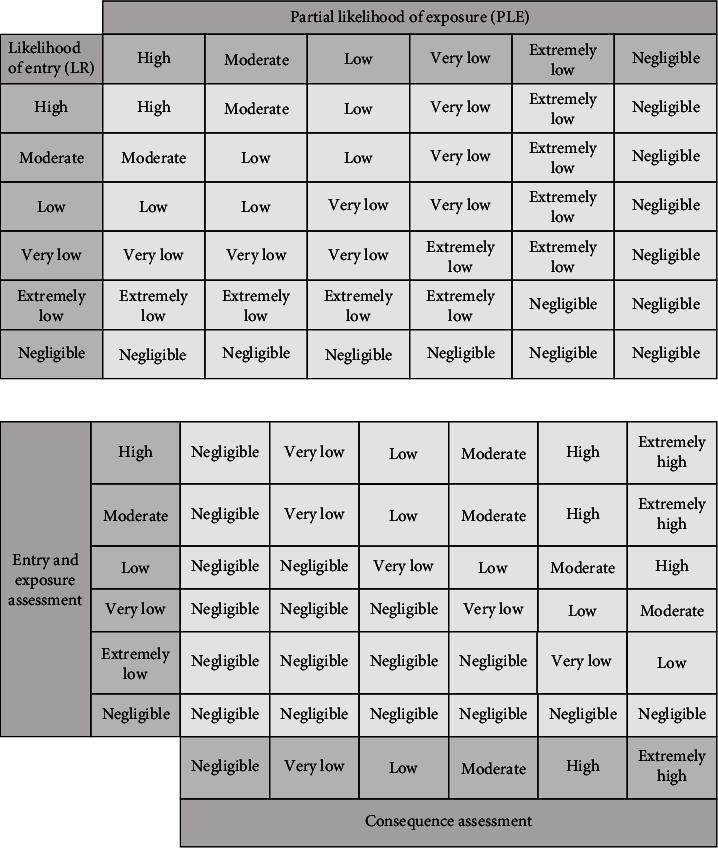
Integration process of the possibilities of entry and exposure assessments (a) and for the risk estimation (b).

Risk estimation is obtained by integrating the results of entry assessment, exposure assessment, and consequence assessment, and the integration process of the possibility is as follows (Table 4 and Figure 3).

### 2.4. Risk Management

Comparison and analysis of the risk estimated in the risk assessment in relation to South Korea's appropriate level of protection (ALOP) to find alternatives to ensure that the assessed risks can be managed in accordance with South Korea's ALOP and implementation of the effectiveness and feasibility in the order of the assessment. When preparing risk management plans, apply applicable biosafety measures that can effectively manage risks that may arise from hazards related to the importation of aquatic organisms (or their products) designated for quarantine purposes as a standard. In addition, the negative impact on international trade should be minimized by limiting it to the minimum level necessary to protect South Korea's aquatic life, the aquatic environment, and public health. Moreover, the impact on the economy, environment, society, and culture due to recommendations and risk management plans of international organizations such as WOAH should be considered. Last, risk management plans should be prepared in consideration of uncertainty, and scientific evidence or logic for the prepared plans should be clearly explained. However, if additional information on uncertainties is confirmed, the prepared risk management plan may be reviewed. In conclusion, the risk management plans are presented as quarantine methods and quarantine standards that can be applied to effectively reduce the risk of DIV1 and minimize the negative impact on international trade.

### 2.5. DIV1

The import risk analysis performed in this study also focused on the risk of DIV1, which may be caused by the following decapods that can be imported into South Korea from all countries. Target species are crayfish, crab, shrimp, and susceptible decapods that may be disease vectors. Purposes of use are human consumption, ornamental, aquaculture, research, and nonhuman consumption (for animal feed or bait). The product conditions considered are live, frozen, and refrigerated, and the direct entry of DIV1 (virus stocks or infected tissues) for research. The aquatic products for “non-human consumption” here refer to the products in the cases where a memorandum of “Import Obligation Compliance” with a pledge that the products will be used only for the designated purposes by indicating “For Feed” or “For Bait” in requirement field of the import documents is submitted to the Korea Customs Service following the provisions of the Customs Act.

## 3. Results

### 3.1. DIV1 Epidemiology

#### 3.1.1. Pathogen Characteristics

The DIV1 is an icosahedral, double-stranded DNA virus, and the average diameter is approximately 150 nm [1–3, 11]. The International Committee on Taxonomy of Viruses classifies DIV1 as the Decapodridovirus genus of the Iridoviridae family [12]. Members of the Iridoviridae family have been reported infecting various species of invertebrates and vertebrates. At least five types of Iridoviruses have been described to infect crustaceans [2, 13–16].

#### 3.1.2. Clinical Signs and Gross Pathology Lesions

DIV1 infection has no specific clinical signs since all the clinical signs described in DIV1 infection have been reported as well for many other viral infections in crustaceans, including white spot syndrome virus (WSSV), infectious hypodermal and hematopoietic necrosis virus (IHHNV), etc. [4, 17]. It has been reported that some individuals of whiteleg shrimp (*P. vannamei*) show reddish body discoloration and that white triangular spots appear on the carapace below the snout in giant freshwater prawn (*M. rosenbergii*) [1, 3, 9]. In addition, in some individuals of giant freshwater prawn (*M. rosenbergii*), turbidity of muscle tissue and damage to antennae were observed [3]. It has also been reported that when shrimp and crayfish were experimentally infected with DIV1, cessation of feeding and lethargy were observed [2].

#### 3.1.3. Target Organs

DIV1 has been reported to mainly infect hematopoietic tissues and hemocytes but also infects antennas, pereiopods, pleopods, uropods, gills, muscles, hepatopancreas, lymphoid organs, antennal glands, and connective tissues [1–3, 7, 18].

#### 3.1.4. Histopathology

Basophilic inclusion bodies were identified in hematopoietic tissue, hepatopancreas, pereiopods, muscle, gills, and hemocytes of whiteleg shrimp (*P. vannamei*) infected with DIV1 [1]. In addition, basophilic inclusions and karyopyknosis were also found in the hepatopancreas of giant freshwater prawn (*M. rosenbergii*) [3]. The histopathological lesions are predominantly observed in the epithelia and hemocytes, as reported [1, 3, 17].

#### 3.1.5. DIV1 Susceptible Species and Their Production

The criteria and species of susceptibility for DIV1 are described in Table 5. According to WOAH Aquatic Animal Health Code Chapter 1.5., if all of the susceptible host Stages 1 and 2 were satisfied, or one of them and two or more of Stage 3 were satisfied, they were considered completely susceptible species (Table 5). In the case of polychaetes used as live feed for whiteleg shrimp (*P. vannamei*) brooders, a nested polymerase chain reaction (nested-PCR) method exist for detecting DIV1 [1, 19]. In the case of *Cladocera* spp., DIV1 was detected using a real-time PCR method from individuals sampled from giant freshwater prawn (*M. rosenbergii*) and red swamp crayfish (*Pro. clarkii*) farms in Jiangsu Province, China, so these species can be regarded as a vector [3, 11].

#### 3.1.6. Geographic Distribution

Countries, where outbreaks of DIV1 have been confirmed so far, are China and Taiwan, and China was first reported in 2014 in Fujian Province (the pathogen name was CQIV infection), and then detected in shrimp in Zhejiang Province in China in 2016 [1, 2]. Subsequently, DIV1 was detected in 11 out of 16 provinces through targeted surveillance by Chinese authorities in 2017–2018 [1, 3, 17]. Recently, infections of DIV1 have been reported in redclaw crayfish (*C. quadricarinatus*), whiteleg shrimp (*P. vannamei*), and giant tiger prawn (*Penaeus monodon*) farms in Taiwan [8, 18, 24–26]. In addition, there is an unofficial report that DIV1 was detected at a very low prevalence rate in Thailand [27], and DIV1 was also detected in wild giant tiger prawn (*P. monodon*) caught in the Indian Ocean in 2020 [18].

#### 3.1.7. Prevalence of DIV1

It has been reported that the prevalence of DIV1 in aquaculture production in China ranges from 0% to 50% [1, 7], and the results of DIV1 surveillance in China for 625 farms in seven provinces from 2014 to 2016, a prevalence of 15.8% (99/625 sites) was confirmed [1]. By species, whiteleg shrimp (*P. vannamei*) accounted for 15.5% (89/575), fleshy prawn (*P. chinensis*) accounted for 15.2% (5/33), giant freshwater prawn (*M. rosenbergii*) accounted for 50% (5/575), and Kuruma prawn (*P. japonicus*) had a prevalence of 0% (0/7) [7]. In the case of Taiwan, DIV1 was detected in one red swamp crayfish (*Pro. clarkii*) farm as a result of active surveillance (21.5.8. to 21.5.21.) of 103 farms in Taiwan. In addition, in Taiwan, whiteleg shrimp (*P. vannamei*), giant tiger prawn (*P. monodon*), giant freshwater shrimp (*M. rosenbergii*), crabs, and crayfish farms are being monitored, and a total of 16 DIV1-positive cases have been reported. Thai researchers confirmed that 5 of 26 (19%) clinically healthy-looking giant tiger prawns (*P. monodon*) collected from the Indian Ocean were infected with DIV1 [18].

#### 3.1.8. Mortality Rates of DIV1

Mortality rates of over 80% have been reported for farmed shrimp and crayfish [1–3]. Furthermore, mortality due to DIV1 infection may be exacerbated by poor water quality and environmental conditions [28, 29]. Mortality rates of 20% in Taiwanese giant tiger prawn (*P. monodon*) farms and 0%, 20%, and 90% mortality rates in three whiteleg shrimp (*P. vannamei*) farms are reported, respectively, and no mortality was observed in 12 redclaw crayfish (*C. quadricarinatus*) farms [8].

#### 3.1.9. Routes of Transmission

Although the natural transmission route and infectious dose of DIV1 remain uncertain, laboratory experiments have demonstrated that oral exposure to DIV1-infected tissues can result in infection [1, 7]. In addition, reports of experiments of DIV1 transmission through feces of DIV1-infected animals and intramuscular injection have been successful [1, 2, 22, 23]. Recently reported in Taiwanese literature, it is speculated that DIV1 was introduced from the contamination of ponds by migratory birds (estimated due to feces) and imported shrimp seed (postlarvae) [25]. It is not yet certain, but like other Iridoviruses, it is possible to transmit through water [3, 30, 31]. The possibility of indirect infection by fomites is difficult to assess due to the lack of information on the biochemical properties of the virus itself, but it can be assumed that it shares characteristics with other similar viruses, such as WSSV. In particular, given that high titration of DIV1 was detected in the hemolymph, antennal flagellum, rostrum, gills, hepatopancreas, pleopods, muscle, and tail, solid or liquid waste may be easily contaminated [3, 11]. DIV1 can be transmitted by vectors such as *Cladocera* spp. or polychaetes, which are common organisms in water ecosystems of South Korea [3, 9, 17, 19, 20, 32].

#### 3.1.10. Diagnostic Methods

DIV1 diagnostic methods include *in situ* hybridization [1], conventional PCR [2], nested-PCR [1], TaqMan probe-based real-time PCR [7, 11], *in situ* DIG-labeling-loop-mediated DNA amplification, and the recombinase polymerase amplification [33] have been developed. The nested-PCR and the real-time PCR are highly sensitive, and their effectiveness has been verified [1, 11].

#### 3.1.11. DIV1 Diagnosis Service Development Status

Currently, Australia's Genics Co., Ltd. provides rapid diagnostic testing services for 14 shrimp diseases, including DIV1, through the Shrimp MultiPath Xtra PCR testing service (https://www.genics.com.au/services/). In addition, Taiwan's GeneReach Corp. develops and sells qPCR reagents for rapid diagnosis of DIV1 (https://www.genereach.com/index.php).

#### 3.1.12. Treatment of DIV1 Infection

There is no treatment known to be effective so far, but it is thought that some comparisons can be made through the following literature [3, 11]. (1) Establishment of a surveillance plan for farms to enhance biological security, quarantine, and DIV1 inspection of brooders or postlarvae [34]. (2) Since infected penaeid shrimp do not have clinical signs (and gross pathology lesions), the presence or absence of contamination is inspected using the characteristic lesions of DIV1 (triangular white spot) in giant freshwater prawn (*M. rosenbergii*) as an indicator. (3) General biosecurity measures (cleaning and disinfection) to minimize transmission through equipment, vehicles, or personnel involved help prevent transmission. (4) The spread of disease can be prevented by limiting the movement of living crustaceans and by quickly removing moribund or dead individuals. (5) Combined farming of several species of crustaceans should be avoided. (6) High-density aquaculture of crustaceans should be avoided, and feed other than live or frozen decapods or polychaetes should be used as feed for brooders, and if necessary, procedures such as disinfection should be followed before feeding [19].

### 3.2. Status of Quarantine Policy in South Korea

The National Fisheries Products Quality Management Service and its affiliated organizations are designated as institutions in charge of the quarantine of imported and exported aquatic organisms, and a person who has received training in quarantine as an aquatic disease inspector or veterinarian is appointed as an aquatic organism quarantine officer. Quarantine of imported and exported aquatic organisms is conducted for aquatic organisms (or their products) designated for quarantine purposes, their containers, packaging, and traveler's belongings, and the scope of designated quarantine objects are described in Table 2. In addition, a person who intends to import aquatic organisms (or their products) designated for quarantine purposes must attach a quarantine certificate issued by the government agency of the exporting country.

### 3.3. Hazard Identification

#### 3.3.1. Backgrounds

*(1) Imported Amounts of DIV1 Susceptible Species and Vectors* Decapods, a group of 10-legged crustaceans belonging to the class Malacostraca, encompass a diverse array of species, including shrimp, lobsters, crayfish, and crabs. The order Decapoda is characterized by the possession of up to 38 appendages arranged in one pair per body segment. These organisms are distributed globally, with an estimated 15,000 extant species spanning approximately 2,700 genera and an additional 3,300 fossil species. Notably, the Korean Peninsula alone harbors an estimated 130 decapod species [35–37]. Representative DIV1-susceptible species produced or naturally inhabiting water systems in South Korea include the Dendrobranchiata species Kuruma prawn (*Penaeus japonicus*), fleshy prawn (*Penaeus chinensis*), and whiteleg shrimp (*P. vannamei*). Species belonging to the Pleocyemata include the brackish water Chinese mitten crab (*Eriocheir sinensis*) and the Oriental river prawn (*Macrobrachium nipponense*). Polychaetes, the vector of DIV1, is currently being imported into South Korea with two species (*Perinereis aibuhitensis* and *Marphysa sanguinea*), according to the National Fisheries Quality Management Service's “Non-human Consumption Aquatic Product Quarantine Management Plan Research (2019–2021).” Among the major susceptible species, Chinese mitten crab (*E. sinensis*), whiteleg shrimp (*P. vannamei*), and Oriental river prawn (*M. nipponense*) are steadily imported, and their total import amount is about 52,000 tons on average (Table 6).

*(2) Domestic Production of Crustaceans* Domestic crustacean production steadily increased from 80,165 tons in 2020–95,896 tons in 2022, and the production value also recorded over 800 billion KRW (₩) on average. Among them, shrimp accounts for 48% of domestic crustacean production and 42% of production value. Domestic production of crabs is on the increase from 38,107 tons in 2020–52,280 tons in 2022, and the production value has also increased from 458 billion KRW (₩) to 476 billion KRW (₩), creating high-added value. The production of crab accounts for 51% of the total production of crustaceans and 58% of the production value (on average). The production of other crustaceans in Korea is about 655 tons on average for 3 years, with a production value of five billion KRW (₩). Whiteleg shrimp (*P. vannamei*), a DIV1 major susceptible species, is one of the highly produced species in Korea, with an annual average of over 9,000 tons and 154 billion KRW (₩) produced (Table 7).

*(3) Domestic Consumption of Crustaceans* Crustaceans are a popular aquatic product with an annual average consumption of 233,000 tons in South Korea, but the self-sufficiency rate is only 38% on average, so it can be seen as dependent on imports (Table 8).

As a result of checking the import weight and amount of domestic crustacean products (Customs Service HS-code: 0306) over the past 3 years since 2020, the domestic crustacean import and export volume, and domestic consumption remains steady (Table 8). Nevertheless, self-efficiency has been steadily increasing every year, which seems to be due to the increase in domestic production (Tables 7 and 8). Among crustaceans, shrimp, shrimp meat, crab (other), snow crab, and lobster accounted for about 20% of all imported aquatic products and were recorded in the top 15 imported items in 2020 (https://www.fips.go.kr).

#### 3.3.2. Hazard Identification of DIV1

DIV1 is a pathogen that has been reported in some major seafood exporting countries (China, Thailand, and Taiwan), and it is highly likely to be introduced into South Korea in frozen, refrigerated, and live aquatic products. DIV1 has not yet been reported in South Korea and has not been detected. In this sense, DIV1 is classified as a hazard, and it is necessary to examine its biological, environmental, and economic (fishery) impacts on susceptible hosts inhabiting natural systems or domestically farmed after entry and exposure in South Korea.

In conclusion, DIV1 could be identified as a hazard due to the following factors: (1) Species susceptible to DIV1 are imported from countries with DIV1 presence, and the susceptible species are included in domestic major aquaculture species and living in wild aquatic systems. (2) DIV1 causes mass mortality in shrimp farms in exporting countries such as China and Taiwan, and DIV1 outbreaks have not been reported in South Korea yet. (3) DIV1 is listed as a WOAH-designated disease, and only some exporting countries are restrictively strengthening DIV1 management.

### 3.4. Risk Assessment

#### 3.4.1. Entry Assessment

*(1) Biological Factors* Susceptible species: DIV1 shows or is highly likely to show susceptibility to various decapod species currently being imported into South Korea or may be highly susceptible to DIV1 infection. To date, 10 species have been identified as naturally susceptible to DIV1 infection, of which most are decapod crustaceans and are currently being imported into South Korea (Table 5). DIV1 susceptibility is not considered to be an age-limited pathogen.

Target organs and stability: DIV1 mainly infects organs and cells of hematopoietic origin, such as lymphoid organs, hematopoietic tissues, and hemocytes, causing systemic infection [1–3, 7, 18]. In addition, it was confirmed that tissues of shrimp infected with DIV1 retain infectivity even at −80°C [1, 7].

Vaccination, prevention, and treatment measures in major exporting countries: to date, no DIV1 vaccine is under research and development. International organizations such as NACA and WOAH recommend strengthening biosecurity and surveillance, as no treatment exists.

Quarantine measures of South Korea: Since August 1, 2020, South Korea has designated DIV1 as a disease subject to management through the Minister of Oceans and Fisheries Notice (G/SPS/N/KOR/692, July 17, 2020) and is conducting quarantine on 10 live decapods and 8 frozen and refrigerated prawn products.

*(2) National Factors* Prevalence and mortality in exporting countries: A prevalence of more than 25% has been reported in farmed whiteleg shrimp (*P. vannamei*) in China [1, 7], and a mortality rate of 20% has been recorded in Taiwan's giant tiger prawn (*P. monodon*). However, few susceptible species are currently imported from Taiwan.

Aquatic organisms' sanitation management institutions in major exporting countries (capacity, surveillance, and sanitation management, localization): Many countries, including China, Taiwan, and Vietnam, have established DIV1 surveillance systems to strengthen disease spread, prevention, and control. So far, 22 countries have conducted pre-export inspections when exporting DIV1-susceptible species.

- Nicaragua, Taiwan ^*∗*^, Malaysia, USA, Myanmar, Bangladesh, Vietnam, Saudi Arabia, Spain, Singapore, Ecuador, Indonesia, Japan, China ^*∗*^, Canada, Colombia, Thailand ^*∗*^, Panama, Peru, Finland, Philippines, Australia ( ^*∗*^Countries with a history of DIV1 outbreak)

*(3) Factors of Aquatic Organisms (or Their Products) Designated for Quarantine Purposes* Crustacean import and quarantine status (including vectors): Of all crustaceans imported during 2020–2022 (average of about 156,000 tons), DIV1 susceptible species imports averaged about 52,000 tons, accounting for about 33% (Tables 6 and 8). However, an average of about 39,000 tons, 73% of DIV1 susceptible species imports, are aquatic organisms intended for nonhuman consumption (prawns or polychaetes) that are not subject to quarantine (Table 6). In addition, about 621 tons of polychaetes (intended as feed for brooders [19]) were detected as DIV1-positive and were imported into South Korea without quarantine as of 2020. Furthermore, frozen and refrigerated crayfish and crabs were also not subjected to quarantine in South Korea, so only general sensory tests by the Ministry of Food and Drug Safety are being performed.

Ease of contamination and pathogen survival rate according to processing method and storage/transportation methods: Frozen decapods are imported in the form of bulk or individual packaging, and if waste is generated in a processing facility without a disinfection treatment or diverted as feed or bait after import, it is high possible that the surrounding water system will be contaminated with DIV1. Live, frozen and refrigerated decapods are likely to retain infective DIV1 particles. However, since no information is available on the viability rate of DIV1 in the nonhost environment, the risk of effluent from storage facilities for living decapods is highly uncertain. Due to the nature of viral pathogens, it is expected that the viability of the virus will be lost if heat treatment is applied, but information on the conditions for inactivating DIV1 is not yet known.

DIV1 quarantine method for designated quarantine objects: Clinical examination and close inspection are performed. The clinical examination makes sure whether swimming and behavior are normal and observes external features such as body color and body shape, and anatomical features such as internal organs. The close inspection follows the Aquatic Animal Disease Diagnosis Guidelines of the National Fisheries Quality Management Service, and if there is no content in the guideline, WOAH's Aquatic Animal Health Code or internationally recognized test methods are used. The current DIV1 quarantine method is the nested-PCR which Qiu et al. [1] registered and used in the aquatic organism disease diagnosis guideline.

*(4) Entry Assessment of DIV1* South Korea imports decapods, including DIV1-susceptible species. Nonetheless, DIV1 may infect decapods other than the susceptible species reported so far. DIV1 caused a high mortality rate of over 80% in shrimp farms in the major exporting country (China), along with the neighboring countries (Taiwan), and it has also been detected in wild shrimp caught in the Indian Ocean [18]. Many countries, such as China, Taiwan, and Vietnam, are building DIV1 monitoring systems. South Korea has designated 10 DIV1 susceptible species (eight shrimp, two crayfish) as the aquatic organisms (or their products) designated for quarantine purposes and began DIV1 quarantine on the live/frozen/refrigerated shrimp and live crayfish from August 1, 2020 (G/SPS/N/KOR/692, July 17, 2020). Crustaceans imported in frozen or refrigerated conditions are less likely to transmit pathogens through breeding water, but in the case of live crustaceans (for human consumption, ornamental, research, and aquaculture), the risk level may vary depending on the amount of discharge of the breeding water. Some literature predicts that transmission through water, like other Iridoviridae, may also occur, and there are no shrimp stocks (specific pathogen-free or other, including juvenile or adult shrimp) showing resistance to DIV1 infection yet.

DIV1 causes a systemic infection, including organs in the cephalothorax. Specific clinical signs are rare (white triangles at the base of rostrum in *M. rosenbergii*), and DIV1 has been detected from asymptomatic wild animals. In addition, as DIV1 can maintain infectivity even at −80°C, it is expected to maintain sufficient pathogenicity during transportation for trade. While DIV1 mostly causes significant damage to shrimp, it is also known to cause significant damage to crayfish (especially redclaw crayfish), and in the case of freshwater crayfish, including redclaw crayfish, imports for ornamental purposes are as high as those for human consumption. In addition, it is possible that Korean researchers may import infected animals/tissues or pathogens for research, and in the case of aquatic organisms for aquaculture, all aquatic organisms are subjected to quarantine in accordance with Article 25 of the Enforcement Regulations of the Aquatic Organism Disease Control Act. However, in the case of polychaetes, which are known as a vector of DIV1 [19], they are currently imported for animal feed or bait and are being imported without a separate quarantine procedure. Based on this background, the risk levels estimated are described in Table 9. As a result of confirming the exposure pathways, it was determined that nonhuman consumption aquatic products were converted to human consumption, but it was not a problem from the point of view of disease transmission. However, it is believed that education and publicity on related contents are necessary.

#### 3.4.2. Exposure Assessment

The exposure scenarios and basis of risk estimation are shown in Table 9. Exposure assessment analyzed the possibility of being infected with the actual domestic susceptible species after DIV1 entered into South Korea for each farm, hatchery, and wild exposure group.

*(1) Biological Factors* Pathogenicity of DIV1: Since DIV1 causes systemic infection, transmission can occur to all exposed groups if processing to inactivate the pathogen is not performed. Horizontal infection of DIV1 occurs, but it is unclear whether fecal-mediated transmission occurs, and since transmission can occur through feeding infected tissue, the risk of disease transmission through feed or bait is relatively high.

DIV1 susceptible species and the status of imported aquatic organisms (or their products): among the major DIV1 susceptible decapods, shrimp are subject to quarantine for live, frozen, and refrigerated products. In the case of crayfish, only the live condition is subject to quarantine, but most of the DIV1 susceptible species imported into South Korea are imported frozen, headless, or boil-cooked. In particular, most of them are imported for human consumption, and the risk of exposure is low because the possibility of maintaining the pathogenicity of DIV1 in the form of removal of the cephalothorax and boil-cooked is very low. Chinese mitten crab (*E. sinensis*), one of the crabs currently mentioned as susceptible to DIV1 (partially), is at high risk of being exposed to natural waters because it is used live as trap bait without quarantine. However, the Chinese mitten crab (*E. sinensis*) has not yet been naturally infected with DIV1 (only laboratory artificial infection has been performed), making it difficult to view it as a completely susceptible species [22, 23].

Vectors of DIV1: DIV1 has the potential to be transmitted by vectors, and cause exists where DIV1 has been detected from *Cladocera* spp. or polychaetes, and these organisms are common in domestic water systems [3, 9, 17, 20, 32].

*(2) National Factors* Domestic habitat decapods: The populations of crayfish are relatively less than that of shrimp or crabs, and among 130 decapod species inhabiting natural water systems in South Korea, DIV1 susceptible species are Oriental river prawn (*M. nipponense*), Kuruma shrimp (*P. japonicus*), and fleshy prawn (*P. chinensis*). Whiteleg shrimp (*P. vannamei*), a major susceptible species, is a major farmed breed in South Korea, and there are about 470 hatcheries and farms nationwide.

Custom and culture: In South Korea, live bait is mainly used as bait, and representatively shrimp, polychaetes (fishing bait), or crabs (trap bait) are used, and crayfish are rarely used [38]. In addition, there is a culture in South Korea that prefers raw feed during fish farming [39], and polychaetes are sometimes fed especially for the sexual maturation of brooder shrimp, Qiu et al. [19] detected DIV1 from these polychaetes. To curb the use of raw feed, as of 2023, a policy for compulsory compound feed is being promoted, but the target breed is limited to olive flounder (*Paralichthys olivaceus*), so it is irrelevant to shrimp.

In South Korea, there are no popular (or traditional) dishes using freshwater crayfish (redclaw crayfish, red swamp crayfish) compared to shrimp or crabs. Currently, most of the freshwater crayfish imported into South Korea are distributed in small packages and are used in dishes such as “mala longxia,” with their shells and organs attached at Chinese restaurants.

*(3) Factors of Aquatic Organisms (or Their Products) Designated for Quarantine Purposes* Among the susceptible species used for human consumption (eight shrimp and two crayfish), all live, frozen, and refrigerated shrimp are under quarantine. Crayfish are subjected to quarantine only in live animals, but 70% of the imported products are imported in the form of boil-cooked, and the total imported amount is less than that of shrimp or crabs, so the risk can be considered low. In addition, since most of them are used as special cooking ingredients, they are distributed or consumed without processing, such as shell removing.

Ornamental decapods susceptible to DIV1 are generally imported live and are subject to quarantine. However, there is a risk of heterogeneous infection in many cases where several species are combined due to the conditions of the aquaria (space constraints). Among large-scale aquaria that directly import ornamental aquatic life from overseas, places with quarantine enforcement sites are managed by the National Fishery Products Quality Management Service to have carcass treatment facilities mandatorily.

In the case of research use, due to systematic sample management and waste disposal procedures in research institutes, even if pathogens are infected, the probability of testing them in advance is high (by self-test), and even if they are infected, the probability of being transmitted to other water systems is low.

In the case of susceptible species for aquaculture, all imported cases are subject to quarantine, and there is no evidence that vertical transmission of DIV1 occurs.

Since most nonhuman consumption uses of imported susceptible species are fishing bait or as animal feed, they are directly exposed to fish farms, hatcheries, and natural water systems. Polychaetes, a major imported variety, are ad DIV1 vector and can damage various susceptible decapod hosts. However, when polychaetes are used for bait, there is a provision regarding the prohibition of unauthorized disposal following Article 7, Paragraph 2 of the Fishing Management and Development Act (minimum fine of 3 million KRW (₩) or less). There is a case in which DIV1 was detected in polychaetes used as live feed for brooders [19], but there is no case of the disease in shrimp caused by polychaetes carrying DIV1. However, since shrimp diseases such as WSSV and IHHNV could have been transmitted through polychaetes, the possibility of DIV1 transmission through polychaetes cannot be ruled out [40, 41].

#### 3.4.3. Integration of Entry and Exposure Assessments

The levels of risk as per integration of the entry and exposure assessments are shown in Table 9.

#### 3.4.4. Result Assessment

Direct results: There is a high likelihood of damage to the shrimp farming industry exists, as whiteleg shrimp (*P. vannamei*) and giant freshwater prawn (*M. rosenbergii*) are the main susceptible species of DIV1 in South Korea. Here, a case of an aquaculture industry of fleshy prawn (*P. chinensis*) collapsed due to the prevalence of white spot syndrome in the past, and whiteleg shrimp (*P. vannamei*) replaced the main aquaculture species. In South Korea, there are about 470 whiteleg shrimp (*P. vannamei*) farms nationwide, but fleshy prawns (*P. chinensis*) and Kuruma prawns (*P. japonicus*) are also produced in the west coast area (https://www.fips.go.kr). Also, according to WOAH [8], the mortality rate of whiteleg shrimp (*P. vannamei*) caused by DIV1 is up to 90%, so huge damage is expected to the shrimp farming industry when DIV1 may enters. In addition, Oriental river prawn (*M. nipponense*), Kuruma prawn (*P. japonicus*), and fleshy prawn (*P. chinensis*) living in natural water systems in South Korea can also be affected by DIV1 diseases. In particular, DIV1 is transmitted by vectors, where *Cladocera* spp. or polychaetes can play a role, and since they are all living organisms in domestic water systems, huge damage may be expected [3, 9, 17, 19, 20, 32].

Indirect results: Costs for disease control, such as farm surveillance and quarantine, increase due to the entry of DIV1. In addition, as a result of examining potential trade losses to South Korea when a DIV1 outbreak is reported, South Korea currently exports shrimp and shrimp products to an average of 34 countries (Ministry of Food and Drug Safety). Although the volume of trade is not large, it is expected to adversely affect the overall trade in aquatic products due to the external image of South Korea's aquatic products. In the past, the negative perception of consumers caused by partial media reports on the detection of WSSV in shrimp in circulation has had a great impact on shrimp consumption, and negative effects are expected on the local economy (shrimp festivals, etc.). Table 9 shows the estimated levels of risk and the summaries.

#### 3.4.5. Risk Estimation

The levels of risk estimated according to the integration process are shown in Table 9.

### 3.5. Risk Management

Risk management plans have been established to maintain the nation's ALOP for DIV1 at the “Very Low” level seen in advanced countries in general, and the details follow WOAH Aquatic Animal Health Code Chapter 5.4. (2022).

Frozen/refrigerated crayfish or crabs: The risk of the decapods for human consumption is estimated to be “Very Low” risk, but in case the natural infection of the two reported crab species (*E. sinensis* and *Pachy. crassipes*) and banana prawns (*P. merguiensis*) is confirmed, they become completely susceptible species, and the new exposure routes of rock crabs (*Pachy. crassipes*) and banana prawns (*P. merguiensis*) is identified, the scope of quarantine targets needs to be expanded through their registration as the aquatic organisms designated for quarantine purposes.

In the case of crayfish, both the reported species are susceptible, but the import volume of the frozen and refrigerated freshwater crayfish is significantly lower than other decapods, and the amount of byproducts and wastes from processing is also estimated to be relatively small, with most of them expected to be consumed for food, however, it is necessary to raise the risk level and include them in the aquatic organisms (or their products) designated for quarantine purposes when new exposure routes are identified, major import types (boil-cooked) change or import volumes increase.

Decapods for nonhuman consumption (for feed or bait): The decapods for feed or bait have been added to the scope of the aquatic organisms (or their products) designated for quarantine purposes for aquaculture, human consumption, ornamental, research purposes, or surveys under Article 23 Paragraph 1 of the Aquatic Organism Disease Control Act, and if the live/frozen/refrigerated products who DIV1 sensitivity has been confirmed are designated aquatic organisms designated for quarantine purposes and quarantined, it is expected that the level of risk that is currently estimated to be at a moderate level can be lowered to a “Very Low” level, which is the appropriate level of protection in South Korea.

Polychaetes (for feed or bait): It is expected that the level of risk of the entry of DIV1 due to polychaetes suspected to be a vector of DIV1 can be lowered to a “Very Low” level by adding the provisions on “Polychaetes for feed or bait” and on the live, frozen, and refrigerated products to Article 25 (Scope of Aquatic Organisms Designated for Quarantine Purposes in the Enforcement Regulations of the Aquatic Organism Disease Control Act). Items and types of imports requiring the application of DIV1 management plans and sanitary conditions are shown in Table 10. In particular, nonhuman consumption decapods and polychaetes are currently subject to high tariff rates of 10%–20%, the same as human consumption. Therefore, it is believed that if the tariff rate is reduced to the same level as other feed fishery products, such as 0%–5%, and designated as a quarantine target item, it will be able to resolve a large part of the opposition from the industry.

## 4. Discussion

In the case of new diseases without data, it is not easy to conduct an import risk analysis based on scientific evidence. In particular, in the case of aquatic animal diseases, research is relatively insufficient compared to animal diseases. That is why it is important to create a system that can systematically control or quarantine various diseases, including emerging ones. From this point of view, in the case of aquatic organism diseases, an institutional approach to management is relatively more important. In particular, if there is no scientific inactivation or disinfection method, institutional methods such as quarantine and import sanitary conditions should be actively sought. Therefore, in the case of DIV1, the target pathogen in this study, it causes high mortality and is a highly contagious pathogen, so it is more urgent to establish preventive measures than curative measures, and this study was conducted to secure scientific grounds for emergency quarantine.

To manage DIV1, Australia sets the risk of DIV1 to “Very low” or lower for three types of items: “head and shell removal,” “cooking,” and “value-added products” [10]. In addition, if there are any suspicious symptoms or unusual matters related to diseases subject to report in Australia, including DIV1, a 24-hr service using operating an emergency disease hotline is available so that the department in charge can quickly handle them (disinfection, treatment, etc.). In order to maintain biosecurity, Taiwan is conducting DIV1 quarantine for imported items of susceptible varieties targeting all countries (WTO/SPS-G/SPS/N/TPKM/531). It must be inspected for DIV1 within 14 days from the date of export to prove disease-free, and there must be no DIV1 outbreak history in the exporting area or facility for at least 2 years. As a special feature, if there is a case where the same importer has previously imported designated quarantine items from the same water area or aquaculture facility and passed quarantine, the quarantine will be exempted once within a month, and random quarantine will be conducted for additional import cases thereafter. Vietnam, which shares a border with China, where DIV1 has been found, recommends preventive measures against the spread of DIV1 in areas adjacent to China and cooperates with local governments, police, and border guards to respond strongly to illegal import, transportation, and sale of shrimp breeds [42].

Quarantine is currently the only and most effective means to prevent the introduction of diseases from abroad. This study mainly focused on lowering the risk of entry through quarantine-related content. This is because other exposure risks that may occur due to the conversion of aquatic products for human consumption to nonhuman consumption (for feed or bait) have not been identified. However, in Australia, there is a case of WSSV transmission caused by human consumption of shrimp [43], so labeling such as “prohibited use other than human consumption” and “prohibited use as fishing bait” is suggested as a risk management measure to reduce this risk, and this method is considered to be an effective management method that can reduce the risk factors of aquatic animal pathogens. However, in this study, no cases of conversion from human consumption to nonhuman consumption were identified. In addition, even if there is such a case since most human-consumption prawns are subject to quarantine, it is necessary to carefully monitor changes in the amount of imported frozen/refrigerated crayfish that are not subject to quarantine and the route of exposure. Furthermore, in the case of incompletely susceptible species such as Chinese mitten crab (*E. sinensis*) and striped shore crab (*Pachy. crassipes*), it is necessary to prepare a management plan if evidence of natural infection or completely susceptible species is obtained later.

The sanitary conditions for imported aquatic products that can spread DIV1 presented in this study are only institutional measures to prove disease-free in quarantine procedures. To effectively prevent the entry of aquatic organism pathogens, it is necessary to propose freezing/refrigeration conditions based on scientific experiments or an effective pathogen inactivation method. In particular, if in-depth research at the national level is supported to present import sanitary conditions based on the inactivation method of pathogens such as WSSV and IHHNV [44–46], the entry of foreign pathogens can be effectively blocked.

In this import risk analysis, it is important to devise an appropriate management plan through communication between stakeholders and scientists. Therefore, it is necessary to collect the opinions (questionnaires, legislative notice, etc.) of the directly regulated persons (aquaculture producers, importers, sellers, etc.), the indirectly regulated persons, and other interested parties through legislative notice, electronic public hearings, etc., and also analyze the appropriateness of regulations and the effectiveness of regulatory enforcement. In addition, it is necessary to provide information on the risks of DIV1 to those engaged in fisheries and general consumers as well as the direct and indirect regulated persons, and in preparation for the future questions or information related to DIV1, there should be a department (staff) in charge so that they can be contacted at any time and such information can be updated in real-time.

Importers, consumers, etc., will inevitably bear the economic burden when a product that has not previously been quarantined is newly designated as a target product. It is the economic cost due to the cost of quarantine inspection and the time required for quarantine. Therefore, to successfully introduce such a system, it is necessary to present an alternative to the management plan. In particular, the tariff reduction proposed in this study can be an excellent alternative, but the HS-code must be separated for the application of tariff rates. Unfortunately, however, since the current policy direction of the Korea Customs Service is to integrate HS-code, it seems necessary to coordinate opinions among related ministries to apply these alternatives.

In this study, we suggest measures to lower the level of DIV1 risk for the decapods that are not quarantined for DIV1 but have a high level of risk, including the risk management measures (processing), restrictions on the imports of high-risk products, requesting the quarantine certificates from exporting countries, etc. The DIV1 risk management and reduction measures proposed in this study may be used as the sanitary conditions for imports in future negotiations on quarantine certificates with exporting countries and may be presented as the submittals when the exporting countries request submission of the scientific evidence related to the products subjects to DIV1 quarantine and quarantine standards. Furthermore, this study may also be used as the scientific standards upon the occurrence of any conflict with the interested parties due to the actual future implementation of the DIV1 risk management and reduction measures proposed in this study.

## Figures and Tables

**Table 1:** The volume and price of imported aquatic products (decapods) in South Korea.

Nation (for sale ^*∗*^)	2020		2021		2022	
	Volume	Price	Volume	Price	Volume	Price
Decapods (total ^*∗∗*^)	52	674	45	711	39	658
China	9	37	7	34	2	11
Vietnam	1	6	1	4	1	6
Indonesia	0.4	5	1	9	0.3	4
Thailand	0.4	2	1	3	1	3
Philippines	0.1	2	0.1	2	0.1	1

^*∗*^In the case of import use, it is based on sales.  ^*∗∗*^Based on frozen, refrigerated, and live conditions (excluding dried, salted, boil-cooked, etc.). Resources: Ministry of Food and Drug Safety. Unit: 1,000 tons and one million USD ($).

**Table 2:** Act and notification about import risk analysis in South Korea.

Items	Contents
Article 23 (Things Designated for Quarantine Purposes) of the Aquatic Organism Disease Control Act	(i)Aquatic animals for transplant (aquaculture), eating, ornament, testing, research, surveys, and aquatic plants (ii) Aquatic organism products that are likely to spread pathogens of a contagious aquatic organism disease (iii) Feed, instruments, water, or other similar things that are likely to spread the pathogens of a contagious aquatic animal disease in the course of transportation or storage of aquatic organisms

Article 25 (Scope of Aquatic Organisms Designated for Quarantine Purposes) of the Enforcement Regulations of the Aquatic Organism Disease Control Act	(i) Aquatic animals for transplant (aquaculture) (ii) Fish, shellfish, crustaceans, amphibians for human consumption, ornamental, and research (iii) Frozen/refrigerated prawns, Haliotidae, and oysters without processing, such as heat treatment and cutting (iv) Aquatic organisms or products (including pathogens or organisms infected with pathogens) that have been approved by the Director of the National Fishery Products Quality Management Service

Article 37 (Import Risk Analysis) of the Aquatic Organism Disease Control Act	(i) If deemed necessary for effectively conducting quarantine inspections, the Minister of Oceans and Fisheries may assess the degree of a risk that the pathogens of a contagious aquatic organism disease transmitted into South Korea are likely to affect aquatic organisms and aquatic environments and may conduct an analysis and assessment on the importation of aquatic organisms, by including the details for lowering the degree of such risk (ii) Matters necessary for the method and procedures for conducting an import risk analysis shall be prescribed by the Ordinance of the Ministry of Oceans and Fisheries

Notification on the Methods and Procedures for the Implementation of Aquatic Organism Import Risk Analysis	(1) Identification of hazards (a) The process of identifying and listing the types of pathogens that can potentially enter into South Korea due to the import of the specific aquatic organisms designated for quarantine purposes, etc. (2) Risk assessment of the aquatic organism disease (a) Qualitative or quantitative assessment of the possibility of entry, settlement, and spread into the country of the hazard through the import of the aquatic organisms designated for quarantine purposes, and the size and result of such effects on biological, environmental, economic, and other areas (3) Risk management of aquatic organism disease (a) Comparison and analysis of the risk estimated in the risk assessment in relation to South Korea's appropriate level of protection (ALOP) to find alternatives to ensure that the assessed risks can be managed in accordance with South Korea's ALOP, and implementation of the effectiveness and feasibility in the order of the assessment

**Table 3:** The risk definition and criteria of assessment following entry pathway and scenarios.

Risk no.	Definition	Criteria of assessment
1	If the disease does not exist in the exporting country	(i) Outbreak reports of exporting countries. (ii) Prevalence of exporting countries (wild and aquaculture)

2	If the disease is controlled by the exporting country or exporter	(i) Disease surveillance (management) system of the exporting countries (or companies) (ii) Geographical connection with major outbreak areas in the exporting countries

3	If the disease is detected in pre-export inspection	(i) Degree of manifestation of gross pathology lesions of infected individuals (ii) Quarantine and inspection system of the exporting countries

4	If the pathogen loses its infectivity during transport	(i) Transportation conditions (temperature, duration, method, etc.) (ii) Pathogen viability according to transportation conditions

5	If the importing country can detect the pathogen	(i) Degree of manifestation of gross pathology lesions of infected individuals (Consider the period of transportation and the condition after the infection has progressed.) (ii) Quarantine and inspection system of the importing country

**Table 4:** Descriptive definitions of likelihoods for assessments.

Entry and exposure assessment		Consequence assessment	
Likelihood	Descriptive definition	Likelihood	Descriptive definition
High	The event would be very likely to occur	Extremely high	Catastrophic
Moderate	The event would occur with an even probability	High	Major
Low	The event would be unlikely to occur	Moderate	Intermediate
Very low	The event would be very unlikely to occur	Low	Low
Extremely low	The event would be extremely unlikely to occur	Very low	Minor
Negligible	The event would almost certainly not occur	Negligible	Insignificant

**Table 5:** The criteria of susceptible species and DIV1 susceptible species.

(a) The criteria of susceptible species of DIV1				
Classification		Criteria	Definition	
Stage 1		(i) Naturally infected species (including outbreaks in aquaculture)	(i) Infections caused by noninvasive and invasive laboratory experiments are not considered	

Stage 2		(i) DIV1 had detected by nested-PCR method of Qiu et al. [1] or real-time PCR method of Qiu et al. [7] in WOAH disease card of DIV1	(i) Must be detected by a globally accepted detection method. (ii) There is no WOAH Aquatic Manual for DIV1 yet	

Stage 3		(1) The pathogen is multiplying in the host, or the pathogen is developing in or on the host (2) A viable pathogen is isolated from a susceptible species presented or transmitted to a pure infective individual (3) Infection results in clinical or pathological changes (4) The specific location of the pathogen matches the expected target tissue	(1) When proving that pathogens exist in the host through analysis such as transmission electron microscopy (TEM) (2) In case of transmission (horizontal infection) from a susceptible species that satisfies (1) of Stage 3, or the disease occurred from a completely natural environment (wild) (3) In the case of the reported clinical or pathological lesions from a susceptible species that satisfies (1) of Stage 3 in the reported susceptible species (4) When the exact target tissue is identified by (1) of Stage 3 and the pathogen is detected from that tissue	

(b) DIV1 susceptible species				
Classification		General name (scientific name)	Basis	References

Completely susceptible	Natural infection	Redclaw crayfish (*Cherax quadricarinatus*)	Stages 1–2 and (1) of Stage 3	[2]
		Oriental river prawn (*Macrobrachium nipponense*)	Stages 1–2 and (2), (3), (4) of Stage 3	[3]
		Giant freshwater prawn (*Macrobrachium rosenbergii*)	Stages 1–2 and (1) of Stage 3	[3]
		Giant tiger prawn (*Penaeus monodon*)	Stages 1–2 and (2), (4) of Stage 3	[8, 18]
		(*Macrobrachium superbum*)^a^	Stages 1–2 and (2), (4) of Stage 3	[3]
		Kuruma prawn (*Penaeus japonicus*)	Stages 1–2 and (2), (4) of Stage 3	[19]
		Fresh prawn (*Penaeus chinensis*)	Stages 1–2 and (2), (4) of Stage 3	[1, 19]
		Polychaetes ^*∗*^	Stages 1–2	[19, 20]
		*Cladocera* spp. ^*∗*^	Stages 1–2	[3, 17]
	Complex infection	Whiteleg shrimp (*Penaeus vannamei*)	Stages 1–2 and (1) of Stage 3	[1, 7]
		Red swamp crayfish (*Procambarus clarkii*)	Stages 1–2 and (2), (3), (4) of Stage 3	[2, 3]
		Oriental prawn (*Exopalaemon carinicauda*)	Stages 1–2 and (2), (4) of Stage 3	[17, 21]

Incompletely susceptible	Experimental infection ^*∗∗*^	Chinese mitten crab (*Eriocheir sinensis*)	Stage 2 and (4) of Stage 3	[22]
		Striped shore crab (*Pachygrapsus crassipes*)	Stage 2 and (4) of Stage 3	[22]
		Banana prawn (*Penaeus merguiensis*)	Stage 2 and (4) of Stage 3	[23]

^*∗*^Vectors.  ^*∗∗*^Intramuscular injection. ^a^Although the number of infected individuals (1 of 1) and copies of the virus (1 × 10–1.18 × 10) are relatively small and lower compared to other susceptible species, the WOAH designation criteria for susceptible species include the number of samples and separate standards for viral copies, etc., so in this study, *M. superbum* is also considered to be a completely susceptible species. PCR, polymerase chain reaction.

**Table 6:** The volume and price of imported aquatic products susceptible to DIV1 in South Korea.

Contents ^*∗*^ (status)	2020		2021		2022	
	Volume	Price	Volume	Price	Volume	Price
Giant tiger prawn (frozen)	–	–	4	30	2	11
Oriental river prawn (frozen)	81	291	3	0	35	129
Giant freshwater crayfish (frozen)	9	0	–	–	19	228
Kuruma prawn (frozen)	0.2	6	0.06	0	–	–
Kuruma prawn (live)	0.06	0	–	–	–	–
Whiteleg shrimp (frozen)	15,699	96,090	14,313	92,186	10,028	68,925
Whiteleg shrimp (refrigerated)	0.3	0	–	–	–	–
Chinese mitten crab (frozen)	196	1,870	243	2,697	252	2,729
Chinese mitten crab (live)	92	996	95	1,150	96	1,025
Banana prawn (frozen)	–	–	1	0	–	–
Freshwater crayfish ^*∗∗*^ (frozen)	161	1,151	109	783	128	1,004
Freshwater crayfish ^*∗∗*^ (live)	0.1	0	–	–	–	–
Nonhuman consumption ^*∗∗∗*^ prawns (frozen or refrigerated)	32,872	275,092	36,962	330,915	44,067	409,749
Polychaetes ^*∗∗∗*^ (frozen or live)	621	8,684	567	8,761	492	7,428
Total	49,732	384,180	52,297	436,522	55,119	491,228

^*∗*^In the case of import use, it is based on sales.  ^*∗∗*^Freshwater crayfish species are unidentified.  ^*∗∗∗*^The two items refer to the statistics of the Korea Customs Service. Resources: Ministry of Food and Drug Safety Korea Customs Service. Unit: Tons and $1,000.

**Table 7:** Domestic production of crustaceans and DIV1 major susceptible species in South Korea.

Contents	2020		2021		2022	
	Volume	Price	Volume	Price	Volume	Price
Crustaceans (total)	80,165	811	92,283	861	95,896	790
Prawns	41,346	347	43,511	367	42,940	309
Crabs	38,107	458	48,196	490	52,280	476
Other crustaceans	712	6	576	5	676	5
Fleshy prawn ^*∗*^ (*P. chinensis*)	79	3	144	4	674	12
Kuruma prawn ^*∗*^ (*P. japonicus*)	67	2	92	3	138	5
Whiteleg shrimp ^*∗*^ (*P. vannamei*)	8,124	139	9,545	154	9,504	168

^*∗*^Major susceptible species. Resources: Ministry of Oceans and Fisheries (https://www.fips.go.kr). Unit: Tons and one billion KRW (₩)

**Table 8:** Domestic consumption, self-sufficiency rate of crustaceans in South Korea.

Contents	2020	2021	2022
Import amounts	158	153	156
Export amounts	12	10	14
Domestic consumption	226	235	237
Self-sufficiency (%)	35.4	39.2	40.4

Resources: Korean statistical information service (KOSIS) and aT Korean agro-fisheries and food trade corporation (aT KATI) Domestic consumption = (Output + Import volume)–Export volume Self-sufficiency = (Output ÷ Domestic consumption) × 100 (%). Unit: 1,000 tons and %.

**Table 9:** Results of risk assessments and risk estimation.

Use	Entry assessment	Summary	Exposure assessment	Summary	Integration of entry and exposure assessment	Result assessment	Summary		Risk estimation
Human consumption	Low	(i) Major susceptible species such as frozen/refrigerated shrimp are currently quarantined, while crabs and crayfish have few completely susceptible species	Low	(i) There are fewer crayfish species in South Korea than shrimp and crab (ii) There are unlikely to be used as ad feed or bait (iii) Most frozen/refrigerated crayfish are imported as boil-cooked products (70% in the case of freshwater crayfish) or sold/consumed as special food ingredients with shells/organs intact (no processing waste)	Very low	Moderate	Direct	(i) There is a high possibility that irreversible damage can be inflicted on shrimp farming, including such major sensitive species as whiteleg shrimp, giant river prawn (ii) In the past, South Korea changed the major species of shrimp farming due to the outbreak of white spot syndrome (iii) There is a high possibility of occurrence of a large number of deaths by causing diseases in Oriental river prawns, fleshy prawn, etc. that live in the nation's water systems (iv) In particular, DIV1 can spread by such vectors as *Cladocera* spp. or polychaetes, resulting in a fatal damage to natural water systems	Very low
Ornamental	Low	(i) Live products for ornamental use are under quarantined	Low	(i) Some of the large aquaria directly importing products with a quarantine area are managed by the National Fishery Products Quality Management Service to mandatorily have a carcass processing facility (Article 32 of the Aquatic Organism Disease Control Act) (ii) There is a possibility of live aquatic animals escaping to the wild and the of unauthorized release by breeders	Very low				Very low
Research	Moderate	(i) If infected tissue is imported from abroad, there is a possibility of multi-infection with DIV1 (ii) Exporting conducted following the submission of the aquaculture/study plan and implementation of quarantine (Article 25 of the Enforcement Regulations of the Aquatic Organism Disease Control Act)	Very low	(i) Research institute's self-inspections, systematic sample management, and waste treatment procedures	Very low				Very low
Aquaculture	Low	(i) These are quarantined as they are imported live, and unlike those for other uses, all imports are subject to close inspection	Very low	(i) Direct exposure to fish farms or hatcheries may occur, but as all are imported with the quarantine certificate issued by the exporting country attached and are subject to quarantine upon arrival in South Korea, exposure routes can be blocked	Very low		Indirect	(i) The entry of DIV1 into the nation can cause an increase in disease control costs, including surveillance and quarantine of farms (ii) Korea exports about 1,400 tons of shrimp and shrimp products to 34 countries on average, and although the trade volume is not large, there is a possibility that South Korea's image in relation to aquatic products may deteriorate, and it can negatively affect the nation's overall trade of aquatic products (iii) In the past, the news on the detection of white spot syndrome virus in shrimps in circulation had a major impact on shrimp consumption due to negative perceptions, and this is expected to have had a negative impact on the economy and consumption of local communities (local shrimp festival, etc.)	Very low
Nonhuman consumption (feed or bait)	High	(i) As these are imported without quarantine procedures, potential damage can be caused to various susceptible hosts as a DIV1 vector	High	(i) Direct exposure of fish farms, hatcheries or natural water systems to DIV1 can occur (ii) There is no separate regulation for the disposal of objects left after use. (iii) It is difficult to expect a reduction in pathogenicity due to freezing and refrigeration	High				Moderate

**Table 10:** DIV1 risk management plans and sanitary conditions for imported aquatic products.

(a) DIV1 management plans			
Plans	Target use	Details	Note
Removal of head and shell	Nonhuman consumption (crustaceans ^*∗*^ and polychaetes ^*∗*^ for feed and bait)	(i) Systemic infection occurs due to hemocyte infection, but major targe tissues are removed	(i) Partial loss of function as feed or bait
Quarantine		(i) Designation of crustaceans ^*∗*^ and polychaetes ^*∗*^ for feed and bait as the aquatic organisms designated for quarantine purposes	(i) Redclaw crayfish and red swamp crayfish have no risk of exposure as these are not used for feed or bait (ii) Quarantine of frozen/refrigerated crayfish should be performed if crayfish imports change, or new exposure routes are discovered (iii) In the case of two types of crabs, if evidence of completely susceptible ins confirmed, they should be designated as quarantine targets

(b) DIV1 sanitary conditions			

Pre-export conditions	Nonhuman consumption (crustaceans ^*∗*^ and polychaetes ^*∗*^ for feed and bait)	(i) Attaching the quarantine certificate issued by the competent authority of the exporting country after a close inspection before export	(i) Proving negative after DIV1 close-up and there are no gross pathology lesions
		or	
		(i) Attaching a disease-free certificate issued by the competent authority of the exporting country certifying that the country is a clean country or clean area	(i) Issuance of an export quarantine certificate certifying that the country has been confirmed as a disease-free country through a surveillance method per WOAH hygiene regulations
and			
Conditions for postarrival import quarantine	Nonhuman consumption (crustaceans ^*∗*^ and polychaetes ^*∗*^ for feed and bait)	(i) Implementation of import quarantine and exemption from quarantine for certain product types	(i) Implementation of import quarantine on completely susceptible species (however, exemption from quarantine for products types that fall under the removal of head and shells)
		or	
		(i) Mooring and quarantine (quarantined during the incubation period of the disease)	(i) Examination of gross pathology lesions during the 2-week acclimatization period and implementation of close inspection when gross pathology lesions appear

(c) Alternations for DIV1 risk management plans			

Reduce the tariff rate	Nonhuman consumption (crustaceans ^*∗*^ and polychaetes ^*∗*^ for feed and bait)	(i) Reduce the current tariff rate of 10%–20%–0%–5%	(i) The cost of quarantine inspection can be offset by lower tariff rates, preventing objections from related industries

^*∗*^DIV1 completely susceptible species only.

## Data Availability

The statistical data used to support the findings of this study are available from the corresponding author upon request.
